# The road to tuberculosis treatment in rural Nepal: A qualitative assessment of 26 journeys

**DOI:** 10.1186/1472-6963-8-7

**Published:** 2008-01-11

**Authors:** Augustinus HA ten Asbroek, Merijn W Bijlsma, Puspha Malla, Binjwala Shrestha, Diana MJ Delnoij

**Affiliations:** 1 Department of Social Medicine J2-216, Academic Medical Center, University of Amsterdam, PO Box 22700, 1100 DE Amsterdam, The Netherlands; 2 Institute of Medical Technology Assessment, Erasmus University Rotterdam, The Netherlands; 3 National Tuberculosis Centre, Thimi, Nepal; 4 Department of Community Medicine and Family Health, Institute of Medicine, Tribhuvan University, Kathmandu, Nepal; 5 Netherlands Institute of Health Services Research NIVEL, The Netherlands

## Abstract

**Background:**

The fact that tuberculosis can be treated with the DOTS strategy (Directly Observed Treatment, Short-course) is not enough to control the disease. Patients have to find their way to tuberculosis treatment first. To better understand the route to tuberculosis treatment in rural Nepal we interviewed twenty-six patients under treatment.

**Methods:**

In semi-structured interviews patients shared their disease history and health seeking behaviour. The analysis focused on the encounters with the health care system before enrolment in the tuberculosis treatment program.

**Results:**

Patient routes often started in the medical shop and led via intricate routes with multiple providers to facilities with higher qualified and more competent staff where tuberculosis was diagnosed. Several factors influenced the route to tuberculosis treatment. Besides known patients factors (such as severity of complaints, the ability to pay for services, availability of services and peer support for choosing a provider) specific health services factors were also identified. These included the perceived quality, costs and service level of a provider, and lack of provider initiated referral. Self referral because of waned trust in the provider was very common. In contrast, once tuberculosis was considered a possible diagnosis, referral to diagnostic testing and tuberculosis treatment was prompt.

**Conclusion:**

Patient routes towards tuberculosis treatment are characterised by self referral and include both private and public health care providers. Once tuberculosis is suspected referral for diagnosis and treatment is prompt. Given the importance of the private practitioners in the patient routes, quality improvement initiatives need to address not only the public sector but the private health care sector as well.

## Background

Tuberculosis causes a high burden of disease world wide especially in low and middle income countries, despite the availability of efficacious treatment [1]. Tuberculosis treatment revolves around the DOTS strategy [2,3]. Based on empirical studies as well as prognostic modelling studies, the strategy is considered to be cost-effective despite considerable investments to start-up DOTS [4,5]. An enduring challenge is how to ensure that tuberculosis patients find their way to tuberculosis treatment. Lack of community awareness and patient knowledge about tuberculosis and its management have been mentioned by other researchers as obstructing factors [6,7]. Besides these patient factors, health services factors may be obstructing the road to tuberculosis treatment as well [8]. In this respect, insufficient human resources, lack of diagnostic facilities are some of the known barriers. As a consequence, patients may spend an unnecessary length of time before reaching tuberculosis treatment, resulting in worse outcomes and an increased risk of disease transmission. Internationally, efforts have been made to improve the general health services for patients with lung diseases, including tuberculosis. One example is WHO's Practical Approach to Lung Health (PAL) [9-11]. PAL aims at improving the skills of general primary health care workers and introduces clinical practice guidelines and an implementation strategy. This also includes improving the adequacy of the referral of suspected tuberculosis cases. PAL was recently evaluated in several countries, including Nepal.

As patients ordinarily seek health care from multiple sources for different reasons, both in western as non-western societies [12-14] it is important to understand their routes through the system. Health care policymakers and quality improvement program-designers need to be informed about how patients use the health care system and whether health system factors influence the enrolment in tuberculosis treatment programs. With this in mind we assessed the patient's narrative of their journey that eventually resulted in tuberculosis treatment. We aim to answer the following questions: "What is the route that these patients took through the health care system, from the moment of their first complaints until the start of tuberculosis treatment?" and "What were their reasons for taking this specific route?"

## Methods

### Setting

The study was conducted in the rural lowland district of Nawalparasi, in the Terrai area of Nepal, bordering India. The Terrai area is predominantly flat, well-accessible and densely populated. The Nepalese health care system can be characterized as a national health system based on the principles of primary health care. In addition, there is a large private health care sector [15]. Besides the allopathic health care system a separate health care system for Ayurvedic medicine exists [16] and treatment from traditional practitioners is widely available. The health care system faces multiple challenges such as lack of financial and human resources and poor general infrastructure [17,18].

Nawalparasi has 601,000 inhabitants, for whom the following governmental health care facilities are available: 1 district hospital in its capitol Parasi, 5 primary health care centres, 8 health posts, 63 sub health posts, and a multitude of private health care facilities: hospitals, clinics, consulting medical officers, health workers and drug retail shops [16]. More and larger facilities can be found in the nearest big city, Butwal, approximately one hour by bus from Parasi. The sub-health posts are the smallest governmental health care facilities and provide basic health care. This includes mother and child health care by a mother and child health worker, and general health care by an auxiliary health worker who is also the in-charge of the facility. Health posts have more and higher educated staff. Primary health care centres are yet another, higher, level of facilities. The one district hospital has one medical doctor who is responsible for clinical care and the management of the hospital and who is also the head of the district health care as a whole. The regional hospital is the referral hospital for several districts and offers outpatient and inpatient services by many specialists.

About 45% of the total population in Nepal is infected with tuberculosis, 60% of them are adults. Active tuberculosis incidence in Nepal is estimated at 180/100,000 inhabitants [19]. According to the 2003/2004 annual report of the Ministry of Health, the national case finding rate was estimated 71% (81% in Nawalparasi), the treatment success rate 88% (86% in Nawalparasi). In 2003/2004, 468 new sputum positive cases were identified in Nawalparasi [16]. Nepal reached the global targets for case finding rate (70%) and treatment success rate (85%) in 2003/2004. Data from 2005 show a case finding rate to 67% indicating the challenges in tuberculosis control [19]. The tuberculosis control program coverage increased from 75% in 2000 to 100% in 2006 [20]. This 'vertical' program has been integrated with the general health care facilities over the past decades. In Nawalparasi the facilities for tuberculosis management include 4 DOTS centres for diagnosis and treatment (located in primary health care centres) as well as 10 DOTS sub-centres (located in health posts) where sputum smears can be prepared and patients receive their medication after a diagnosis at a DOTS centre [21]. Patients presenting in primary care health facilities and suspected to have tuberculosis, need referral to a DOTS centre for re-assessment, diagnosis and treatment. Tuberculosis treatment consists of two months intensive treatment under direct observation and six months treatment in continuation phase. Treatment is free of charge. The private health care providers are encouraged to refer tuberculosis suspect patients to the national tuberculosis program. This study was conducted with approval from, and under supervision of the Nepal Health Research Council. This study was embedded in the evaluation of Practical Approach to Lung health for which ethical approval was granted by ethics committees in the U.S. and Nepal. Data for this study were collected between November 2002 and April 2003.The exchange rate for Nepalese Rupees was 78 to 1 US Dollar, 81 to 1 Euro [source: Nepal Rastra Bank] The estimated gross domestic product per capita (purchasing power parity basis) was $ 1400 [22]

### Study population and sampling

Eligible patients were all tuberculosis patients who collected their daily or weekly doses of tuberculosis treatment at one of five selected DOTS (sub-) centres in Nawalparasi on a day that interviews were held. The centres were selected on geographical accessibility (within 2 hours travelling from our base in the district capital) and facility level (two DOTS sub-centres in Health Posts (HP's), two DOTS centres in Primary Healthcare Centres (PHCC's) and one separate DOTS centre in the district capital). The facilities included three remote centres and two more centrally located centres. Each facility was visited on two or three occasions and included always one market day. There were 12 interview days. Patients were invited to participate in the study upon arrival at the DOTS facility in order of arrival. To avoid interference with and from health workers, the consenting patients were interviewed in a separate room, or outside the treatment facility building but mostly on the facilities' premises. We aimed for equal distribution of men and women in the study sample as women are reported to be under represented in the tuberculosis program [23]. Respondents gave informed consent. Anonymity was assured and we explained respondents that participation would not affect their treatment. None of the invited tuberculosis patients declined participation.

### Data collection methods

In semi-structured interviews, respondents were invited to tell their story of how they first perceived their complaints, sought treatment, and eventually ended up in the tuberculosis treatment program. We asked respondents about their route through the health care system and the reasons for this route. A topic list was used to semi-structure the interviews. After brief introductions the respondent was asked to share the story of his/her illness: *What were the initial complaints *when he/she first fell ill? Did he/she get *any help or advice*? *From whom/where *did he/she get help or advice? *Why *did he/she choose this person/provider? *What happened *next? These questions were repeated until the story of how he/she got enrolled in tuberculosis treatment was completed. The interview ended with registering demographic characteristics such as age, gender, profession, position in household, marital status, town or village of residence.

Interviewers (MB, AtA) used one interpreter to communicate with respondents in local languages (Nepali and Bojphuri). Interviews were recorded on tape and summaries of the interviews were written in English by the interpreter and reviewed by the interviewers. To assess the validity of the summaries we transcribed the recordings of five interviews. Independent translators translated these transcriptions from local languages into English. We compared these transcriptions to the written summaries. We found the summaries to correctly reflect the fully transcribed interviews with respect to the routes and reasons for these routes. Details about locations of providers and types of treatment were omitted in the summaries, as were courtesy introductions.

The information from the interview summaries is phrased in third person, reflecting the translations by the interpreter. Information from the five verbatim interviews is presented as quotes. Text in [rectangular brackets] indicates additional information by the researchers about facilities or providers.

### Analysis

The analysis of the interviews consisted of three steps. First, we prepared the data for analysis by marking the text in the interview summaries and transcriptions that referred to: 1. physical complaints, 2. health care providers, and 3. reasons for patient action.

Secondly, we grouped all the words indicating health care providers and used a deductive approach to categorize them. As we aimed to identify the importance of those providers who are often targeted in quality improvement interventions such as WHO's PAL initiative we classified providers according to the services offered (medical consultation or no medical consultation), their anticipated qualifications (health worker or medical doctor) and practice organization (single provider practice or multi-provider practice such as a health post or hospital). Additionally, we indicated private and governmental providers and facilities.

This categorization resulted in three levels of qualifications. Level (A) consists of drug retailers, referred to as medical shops, where drugs can be purchased and where medical consultation or physical examination is commonly not provided. The second level (B) consists of trained health workers who provide consultations and disease management. This includes the staff at sub health posts, health posts and primary health care centres, but also trained health workers with a private practice. Traditional practitioners are included in this level. Lastly, level (C) consists of medical doctors in private practices and hospitals where medical doctors are available for patient care. This framework describes the routes through the health care system to tuberculosis treatment.

Thirdly, we used an inductive approach to analyse the reasons for patient action. We listed all arguments mentioned and grouped these in the following order: Firstly, the initial complaints and providers visited to address these complaints? Secondly, if patients changed providers what were the grounds for this change and how did they choose a new provider? Arguments for not going to a certain provider were also included in the analysis. We did not define categories of reasons prior to the analysis. However, we did create a category enrolment in the tuberculosis programme to allow us to specifically look at the endpoint in the patient journeys.

## Results

We interviewed 26 tuberculosis patients, 12 women aged between 7 and 48 years (mean 29) and 14 men aged between 15 and 74 years of age (mean 42). The 7 year old girl was accompanied by her father who responded to the questions by the interviewer. The adult women had not received any education and worked predominantly in the household. Men were better educated, although only four had completed six years of primary education, one of these four had completed secondary education (see Table 1).

**Table 1:** Respondents' characteristics

***Respondents' characteristics***	***Women (n = 12)***	***Men (n = 14)***
**Age (years)**		
< 10	1	-
10–20	1	1
21–30	6	2
31–40	1	5
41–50	3	2
> 50	-	4
		
**Occupation**		
Housewife	9	-
Unskilled manual worker (incl farmers)	2	7
Skilled manual worker	-	5
Student	1	1
Other (retired from army)	-	1
		
**Education (years)**		
Nil	9	6
1–4	2	1
5 (primary school completed)	-	2
6–11	-	3
12 (secondary school completed)	-	1
Missing	1	1

### General description of routes

The most common starting point for the patient's route was level A, the medical shop, where drugs were purchased with or without consultation from a health worker (10/26). As complaints persisted or even worsened, the routes of our tuberculosis patients led to health workers or medical doctors who held formal consultations. Subsequently, facilities with a good reputation for competence and or diagnostic facilities were consulted at increased financial costs. Eventually the patients arrived higher up in the organizational hierarchy of the health care system.

**Figure 1:**
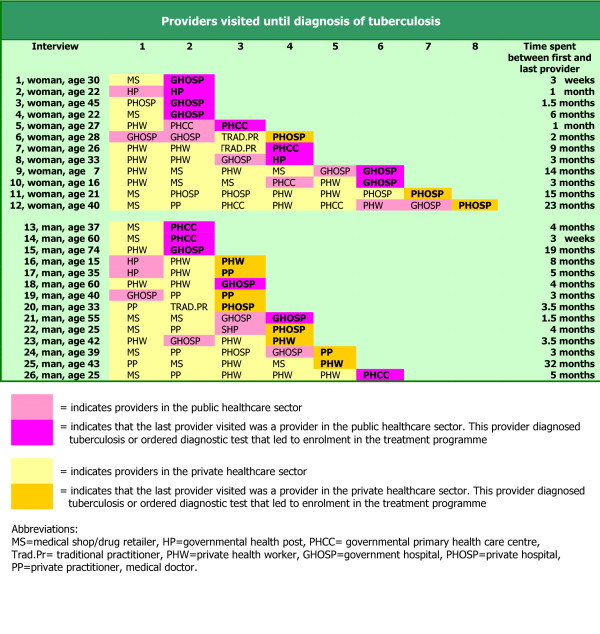
Summary of patient journeys: types of providers visited and time spent beween first and last provider.

When explaining their road to lung health, 21 patients reported that they first went to a private health facility, 5 consulted a governmental primary health care facility at the start of their pathway (see Figure 1). After a minimum of two (2) and a maximum of eight (8) different providers in all possible combinations and orders the patients had reached a provider (11 of level B and 15 of level C) who diagnosed tuberculosis and were subsequently enrolled in tuberculosis treatment. Men appeared to have visited fewer providers than women The last health care facility before enrolment was for 15 patients a government health care facility (i.e. hospital, primary health care facility, or health post), and for 11 patient the last stop was at a private health care facility. The last provider visited by men was more often in the private sector (8/14) than for women (3/12). Overall, patients visited facilities with higher qualified staff at the end of their routes. The routes differed considerably both in length (3 weeks to 2.5 years) as in providers visited.

### Reasons for the followed route

#### Initial complaints, care seeking actions and first providers visited

Respondents' stories started with a variety of complaints, such as fever, cough, headache, blood in sputum, abdominal pain, and loose stools. Most respondents mentioned the complaints to their family members or to others who were close to them. Also the health care options were discussed with them. The first health care seeking actions, following the onset of the initial complaints, were related to the perceived seriousness of illness, anticipated level of competence needed from a provider as well as economic considerations, the reputation and anticipated quality and service level of a provider:

When respondents thought that the complaints were due to a not-severe and not chronic illness they were hopeful that they could manage on their own and bought medication at a medical shop.

He first thought he had the common cold and stayed at home for 2 to 3 days. After 2 to 3 days his nephew bought one tablet for the fever from a medical shop that he took. (Interview summary 14, man, age 60)

Because she thought she was suffering from a simple illness, she did not think it necessary to go to a health post, which in her opinion is for more severe diseases. She first went to a medical shop and got medication for her fever and her headaches. (interview summary 1, woman, age 30).

"At first the gland in the neck was very small ... I did not know what this could be and bought pain medication from medical shops." (interview transcript 4, woman, age 22)

When mentioning reasons for consulting (or not consulting) a specific provider, respondents reported economical factors, the reputation and perceived quality of the provider:

They could not afford to send him to Parasi hospital until six or seven months later. (Interview summary 16, man, age 15)

Someone from her neighbourhood had told her about video X-ray, she knew that she could go to Butwal for this, but she had not enough money. Because she still thought it might be malaria she went to the nearest health post to get a malaria test. (Interview summary 8, woman, age 33)

Many people advised her to go to a good doctor in Parasi or in Butwal or to go to Harnata [location of a famous doctor in India]. The transportation by train and accommodation costs to India were Rs 400. Because she had no money she did not follow up on this advice. (Interview summary 8, woman, age 33)

After this he went to a certain private clinic because his friends suggested he (should) go there and he had heard about this place before. (Interview summary 15, man, age 74)

...and after four days took her to a private health worker at a nearby village in India, 6 km from their home. This health worker was famous in his locality, and he thought that his daughter could get good treatment there. He generally goes there first for treatment if anybody in his family becomes ill. If this does not help he goes somewhere else. (Interview summary 9, girl, age 7)

Some respondents specifically mentioned the service level at sub health posts and health posts.

You have to queue at sub health posts and health posts, and you have to pay five rupees to make a ticket and you can not buy on credit, (Interview summary 4, woman, age 22); "these sub-health posts do not have all the medicines," (Interview transcript 9, girl, age 7); he had to buy medication anyway, he could just as well go to a private health worker immediately (Interview summary 13, man, age 37), are arguments that made respondents reluctant to go to these facilities.

#### Changing providers

All respondents reported to have consulted more than one provider. The main reason was that after the initial treatment the complaints persisted or returned after initial relief. The trust in the provider had waned:

The first and second time he was prescribed medicine for simple cough and fever. He took these for seven days both times. It gave him some relief for some days but it did not cure his cough and after the second time he became angry and said to the medical doctor: if you can cure me then do, if not please tell me so. (....) The third time he was told it was typhoid and was given ayurvedic medicine. This also did not help. The fourth time the same doctor prescribed two medicines for typhoid. He took one that cost Rs 45 per day, it made his muscles shrink and his condition worse. After this the doctor wanted to check his urine and stool but he decided to go to a private nursing home in Butwal. (Interview summary 20, man, age 33)

To consult a different provider was mostly an initiative from the patient or his/her peers, not from the current provider.

He did not get better and his family members and people from his neighbourhood advised him to go to (...). (Interview summary 14, man, age 60)

"It seemed in the beginning that her problem of diarrhoea was controlled when she took medicines. Afterwards, when she got seriously ill, then everybody suggested to take her to Butwal." And "there -in the medical shop- was also a doctor who was a quack. They treated but nothing improved. When the baby got weaker, than I hastened to Butwal." (Interview transcript nr 9, girl, age 7)

(...) when blood started to appear in his sputum he became afraid (...) and someone in his neighbourhood suggested that he was not getting better and advised him to go to (...) (Interview summary 25, man, age 43)

The shopkeeper sold him some medication for his fever, which he took but that did not cure him. He returned and the medical shopkeeper told him he was suffering from typhoid and prescribed medication which again did not make him any better. The third time he asked the shopkeeper if he could cure him or not. The medical shopkeeper now prescribed three injections, one per day for three consecutive days. This made him feel better for three to four days and he went back to work, but afterwards his fever returned and his body felt week. He spent a total of Rs1750 at the medical shop. He decided to go elsewhere. (Interview summary 13, man, age 37)

Someone from her neighbourhood, who had dropped in on the family to watch television, told her mother that her daughter might have tuberculosis because she had been coughing for a long time, and advised to take her to Semari [the nearest Primary Health Care Centre]. He had taken his wife there when she had been coughing, and she had been diagnosed with tuberculosis. (Interview summary 7, woman, age 26)

There was one exception: a respondent mentioned that it was actually the provider that advised her to go to a different provider who had higher qualifications:

... blood in her sputum for four times came. Initially she took medicine from the medical shop near her home. However, she did not get better and her condition became more severe. Then the medical shopkeeper told her that "I cannot control it" and advised her to go to AMDA [a private not-for-profit clinic] in Butwal. (Interview summary 11, woman, age 21)

#### Enrolment in the tuberculosis treatment program

Once a provider suspected tuberculosis, sputum tests were ordered and after the diagnosis of tuberculosis a referral to the tuberculosis programme followed.

After a week of taking medication she went back to the government health post. This time she did tell about her husband's tuberculosis. The health worker examined her, she was given plastic containers and asked to bring sputum to health post the next day. Slides were prepared and these confirmed that she had tuberculosis. (Interview summary 2, woman, age 22)

The first time he went to this health worker he told him he had loss of appetite. The Private health worker listened to his lungs and prescribed syrups and pills costing Rs 900. The second time the same thing happened. The third time he was advised to get a sputum test, he did not turn in enough sputum and tuberculosis was suspected, but not confirmed. The private health worker again prescribed medication. The fourth time his sputum was successfully tested and tuberculosis confirmed and he was prescribed medication and referred to the DOTS centre at the district hospital by this private health worker. (Interview summary 23, man, age 42)

Providers informed diagnosed patients that tuberculosis treatment was available for free:

The medical shopkeepers told him that he could buy it from them, but that he could also get this tuberculosis medication for free at the district hospital. (Interview summary 9, girl, age 7)

It was confirmed that he had tuberculosis and the doctor told him so. He informed him that he should take medicine for 6 months and that he could buy medicine at medicine shops, or get it for free at a government health facility. (Interview summary 20, man, age 33).

Although an exception was also reported:

The next time after seeing the results, the doctor confirmed that he had tuberculosis, told him so and prescribed medication. While prescribing, his brother, who was with him at the consultation, interjected that he could get this medication for free at the health post. The doctor confirmed this. (Interview summary 25, man, age 43)

## Discussion

Our qualitative analysis of twenty-six interviews with diagnosed and treated tuberculosis patients in rural Nepal shows a route that led via multiple providers, in private and public health care facilities, to tuberculosis treatment. The reasons for seeking health care and for their choice of a specific provider were a heterogeneous mix of arguments. These arguments included both patient specific and provider specific arguments. The former included perceived seriousness of the disease by the patient or his peers, the ability to pay for services, and peer support for choosing and visiting a provider. These findings confirm findings of other studies in different contexts in developing as well as developed countries [12-14,24]. Provider specific aspects were also explicitly mentioned by the respondents. These included the perceived quality, costs and service of a provider and being adequately referred by one provider to another. Once tuberculosis was diagnosed the enrolment into the treatment program was prompt and the drugs were known to be free of cost. As this study did not focus on the tuberculosis treatment program but on the *road to *treatment, these findings are informative about the functioning and use of the health care system in general, rather than of the functioning of the tuberculosis treatment program.

A potential problem in our analysis was misclassification of the provider and health care facilities. Whether or not a shop keeper was also a health worker was often unknown to the patient. Likewise, a private health worker was sometimes known to be a health worker or a medical doctor but not always. Although much attention was given to verify the level of providers this might have led to misclassification. As both health workers and medical doctors are mentioned in all phases of the journey we believe that misclassification has not led to distortion of our overall findings. Misclassification of health care facilities is much less likely as this could be verified on the basis of the reported location.

One of the limitations of our explorative and retrospective study is the fact that we only interviewed patients that found their way to tuberculosis treatment. We have no information therefore about the extent to which the routes of these tuberculosis patients are representative for the routes of those who have other diseases or those tuberculosis patients that did not find their way to the national treatment program. Our findings would benefit from a comparison with data from patients with a variety of chronic symptoms and complaints that were actually followed during their journey through the health care system. Our study findings can suggest, however, follow-up studies with a prospective design of some of specific characteristics of the health care system such as the referral process and practices.

For our study we used a convenience sample consisting of 12 women and 14 men who were receiving tuberculosis treatment at one of five DOTS centres. Three centres were remotely located and two more centrally and can be considered representative for all DOTS centres in this district. The adult women were all illiterate, and only four men had completed primary school. Although not representative, this is indicative for the literacy situation in Nepal where literacy among men (62.7%) is much higher compared to women (34.9%) [23]. Also, the diagnosis of tuberculosis in men was more often made by private providers than in women. As consultations with private providers are more costly compared to public providers, this finding may indicate the control over financial resources by men.

Our sample includes only those patients that happened to collect their tuberculosis medication on one of the two to three days that the study was conducted in each of the DOTS centres. This reduced the probability for inclusion of tuberculosis patients that were in the continuation phase of treatment phase when daily visits to the DOTS centre are obligatory. As we focused the study on the patient routes through the primary health care system until enrolment in the tuberculosis treatment program we do not think that this aspect of the sampling strategy affected the findings.

Self-referral because of lack of treatment results and waned trust was very important in the patients' routes. Patients reported that the decision to change provider was usually taken by the patient themselves, supported by suggestions of their peers. Referral by providers seemingly was not based on the provider's perception of his own competence, the symptoms presented by the patient, or the lack of treatment results. In contrast, however, once a provider had thought of tuberculosis, and sputum tests had confirmed the presence of *M. tuberculosis*, referral to the tuberculosis programme seemed a logical and prompt next step. Providers did inform the patient about the fact that tuberculosis drugs are free and that treatment is available near home. Referral to the tuberculosis programme seems to be perceived by the provider as a successful and acceptable end of the consultation and diagnostic process.

The fact that only one health worker referred the patient to a higher qualified provider may indicate that it is difficult for providers to acknowledge that someone else might be more competent to deal with the health problem presented. There could also be a financial reason for the lack of provider-initiated referrals because a provider looses a paying customer when he refers a patient.

Initiatives that aim to strengthen the capacity of primary care facilities, such as WHO's Practical Approach to Lung Health (PAL) [9-11] can benefit from the findings of our study. These narratives highlight the importance of the private sector and the mechanisms influencing the pathways through the health care system. If, for example, sub health posts are expected to become a major tool in passive case-finding of tuberculosis then the perceived low service quality of sub health posts needs to be addressed. In order to improve health care in general – and passive case finding for tuberculosis in particular – the issue of referral needs explicit attention. The review approach that is currently used in the national tuberculosis program may provide useful suggestions: in regular meetings program coordinators present the results of tuberculosis treatment in their areas [25], and discuss issues about quality improvement. Whether a peer review approach can successfully be expanded to include public and private primary care health workers needs further exploration.

## Conclusion

Tuberculosis patients in rural Nepal have followed intricate routes via public and private health care providers before reaching tuberculosis treatment. Self-referral, because of lack of treatment results and waned trust, was the most common reason for changing providers. This not only indicates that providers often fail to treat the patient satisfactorily, but also do they not guide the patient on his/her route to better treatment. Once tuberculosis was diagnosed referral to the tuberculosis treatment program was prompt. These findings on the functioning and use of the general health care system show the importance of quality improvement initiatives. To smoothen the road to tuberculosis treatment these initiatives should not only target the public health care sector but also the private health care sector as patients seek help from both.

## Competing interests

The author(s) declare that they have no competing interests.

## Authors' contributions

AtA made substantial contributions to conception and design, analysis and interpretation of data, and drafted the manuscript. MB contributed to the conception and design of the study, carried the interviews and drafted the manuscript. PM contributed to study design and revised the manuscript critically for intellectual content; BS contributed to the analysis and interpretation of data and revised the manuscript critically for intellectual content. DD made substantial contributions to conception and design of the study, interpretation of data, and revised the manuscript critically for intellectual content. All authors read and approved the final manuscript.

## Pre-publication history

The pre-publication history for this paper can be accessed here:


